# Protection Motivation Theory in Predicting Intention to Engage in Protective Behaviors against *Schistosomiasis* among Middle School Students in Rural China

**DOI:** 10.1371/journal.pntd.0003246

**Published:** 2014-10-16

**Authors:** Han Xiao, Shiyue Li, Xinguang Chen, Bin Yu, Mengting Gao, Hong Yan, Chukwuemeka N. Okafor

**Affiliations:** 1 School of Public Health, Wuhan University, Wuhan, Hubei, China; 2 Xuanwu Hospital, Capital Medical University, Beijing, China; 3 Global Health Institute, Wuhan University, Wuhan, Hubei, China; 4 Department of Epidemiology, University of Florida, Gainesville, Florida, United States of America; Texas Children's Hospital, National School of Tropical Medicine, United States of America

## Abstract

**Background:**

Among millions of people who suffer from *schistosomiasis* in China, adolescents are at increased risk to be infected. However, **t**here is a lack of theory-guided behavioral prevention intervention programs to protect these adolescents. This study attempted to apply the Protection Motivation Theory (PMT) in predicting intentions to engage in protective behaviors against *schistosomiasis* infection.

**Methods:**

The participants were selected using the stratified cluster sampling method. Survey data were collected using anonymous self-reported questionnaire. The advanced structural equation modeling (SEM) method was utilized to assess the complex relationship among *schistosomiasis* knowledge, previous risk exposure and protective measures in predicting intentions to engage in protective behavior through the PMT constructs.

**Principal Findings:**

Approximately 70% of participants reported they were always aware of *schistosomiasis* before exposure to water with endemic *schistosomiasis*, 6% of the participants reported frequency of weekly or monthly prior exposure to snail-conditioned water. 74% of participants reported having always engaged in protective behaviors in the past three months. Approximately 7% were unlikely or very unlikely to avoid contact with snail-conditioned water, and to use protective behaviors before exposure. Results from SEM analysis indicated that both *schistosomiasis* knowledge and prior exposure to *schistosomiasis* were indirectly related to behavior intentions through intrinsic rewards and self-efficacy; prior protective behaviors were indirectly related to behavior intentions through severity, intrinsic rewards and self-efficacy, while awareness had an indirect relationship with behavior intentions through self-efficacy. Among the seven PMT constructs, severity, intrinsic rewards and self-efficacy were significantly associated with behavior intentions.

**Conclusions:**

The PMT can be used to predict the intention to engage in protective behaviors against *schistosomiasis*. *Schistosomiasis* intervention programs should focus on the severity, intrinsic rewards and self-efficacy of protection motivation, and also increase the awareness of infection, and enrich the contents of *schistosomiasis* education.

## Author Summary

In China, millions of population suffer from *schistosomiasis* infection and adolescents tend to have higher infection rates than adults. The Knowledge-Attitude-Practice (KAP) Theory has traditionally been used as guidance to *schistosomiasis* prevention in China. However, despite increases in knowledge among residents in the epidemic areas due to KAP theory-based *schistosomiasis* health education, no significant reduction in water-contact behavior was evident. Therefore, it is of crucial importance to seek alternative health behavior change theories/models that are more effective than the KAP theory to promote purposeful behavioral change. The Protection Motivation Theory (PMT) as a social cognitive model may be an alternative to the KAP theory. In this study, we found that the PMT can be used to predict intention to engage in protective behaviors against *schistosomiasis* infection among middle school students in rural China. Based on the PMT, in addition to enhancing awareness of *schistosomiasis* infection, intervention programs should focus on the severity, intrinsic rewards and self-efficacy of protection motivation.

## Introduction

Prevention of *schistosomiasis* has been a significant public health challenge in tropical and subtropical regions of the world. *Schistosomiasis* epidemic has been recorded in 80 countries, and more than 240 million people are infected and 700 million are susceptible [1], [2]. The global disability-adjusted life years (DALY) attributed to *schistosomiasis* exceeded 70 million in 2008 [3]. Data collected in China in 2011 reported that 286,836 people were infected with *schistosomiasis*, and 30,028 patients were diagnosed with advanced *schistosomiasis*. A recent report estimated that about 372, 644.10 hectares of areas in China was infested with *Oncomelania* snail, a species of snail known to be the primary vector for s*chistosomiasis* [4].

Because of the lack of the acquired immunity, adolescents tend to have higher infection rates than adults [5], [6]. One study in Uganda found that the prevalence rate and the severity of *schistosomiasis* infection often peaked between 10 and 14 years of age and then declines with increasing age [7]. This results are consistent with reports from South Sudan where children aged 10–14 years have the highest infection rate, in addition, school-aged children (6–15 years) were being more likely to be infected than those who are older than 15 years [8]. Furthermore, a longitudinal study in China revealed that despite treatment with *praziquantel*, re-infection rates also remain high among children and young adolescents [9]. The national survey conducted in China in 2006 indicated that 49.3% of acute *schistosomiasis* infection were among those who were 7–18 years of age [10].

The spread of *schistosomiasis* is essentially behavior-related, because humans can only be infected via contact with snail-conditioned water [11]. Many environmental and socioeconomic conditions can influence the spread of *schistosomiasis*, such as raining climate, playing in the water as part of children's daily activities, agricultural economy exposing parents and children in infested water, lack of education and income, however the most modifiable risk factor for *schistosomiasis* infection is behavior [12]–[14].

Since 2005, rigorous and comprehensive measures have been taken to control the epidemic of *schistosomiasis* in China, including the control of snails and snail-conditioned water, improving sanitation by supplying tap water, replacement of bovine with machines for farming, and patient treatment [15]–[17]. However, some challenges still exist for the implementation of these measures in some rural areas, such as farm land unsuitable for tractor farming, economical considerations of bovines beyond their use in farming and low drug efficacy [18], [19]. Therefore, in this case, behavioral prevention such as avoiding contact with snail-conditioned water or taking protective measures is much more important for blocking the transmission of *schistosomiasis*. However, behavioral prevention has not been emphasized, particular in adolescents. An observational study in rural Brazil indicated that among 160 children participants, 96 had contacted with water in a week (3.2 contacts per person per day), and 64% of the contact where in snail-conditioned water [12]. Furthermore, a study in Zimbabwe reported that 87.9% of students do not know the appropriate protective behavioral measures for shcistosomiasis prevention [20]. One study along the *Yangtze River* in China indicated that among young fishermen, 56.8% do not use protective measures correctly [21]. Findings from these studies strongly suggest the need to enhance behavioral preventive measures in the population of adolescent.

The Knowledge-Attitude-Practice (KAP) Theory has traditionally been used as guidance to *schistosomiasis* prevention in China. This approach emphasizes the knowledge of, correct attitudes towards *schistosomiasis* and practical skills to reduce the likelihood of making contact with snail-conditioned water [22]–[26]. One experimental study indicated that receiving a health education program reduced contact with snail-conditioned water from 14.5% at the baseline to 1.8% at one-year follow-up, and the corresponding infection rate from 13.9% to 2.2% among students in a heavy epidemic rural area around the *Poyang Lake* [22]. However, findings in this study have never been repeated. On the contrary, reported studies frequently show that numerous people who have adequate knowledge of and correct attitudes toward *schistosomiasis* do not engage in any protective behavior [23]–[29]. Furthermore, study findings also reveal that despite increases in knowledge among residents in the epidemic areas due to KAP theory-based *schistosomiasis* health education, no significant reduction in water-contact behavior and *schistosomiasis* infection have been evident [28], [29].

The phenomenon of “knowledge-practice” separation [30], [31] described above encouraged many researchers to seek for alternative theories/models that are more effective than the KAP theory to promote purposeful behavioral change. Some researchers suggest that there is an imbalance between knowledge accumulation and behavioral change; therefore increases in knowledge alone may not be adequate to change a person's attitude and behaviors [32]. In addition, cognitive process may play a key role in the process of decision-making, leading to behavioral change [31].

**Figure 1:**
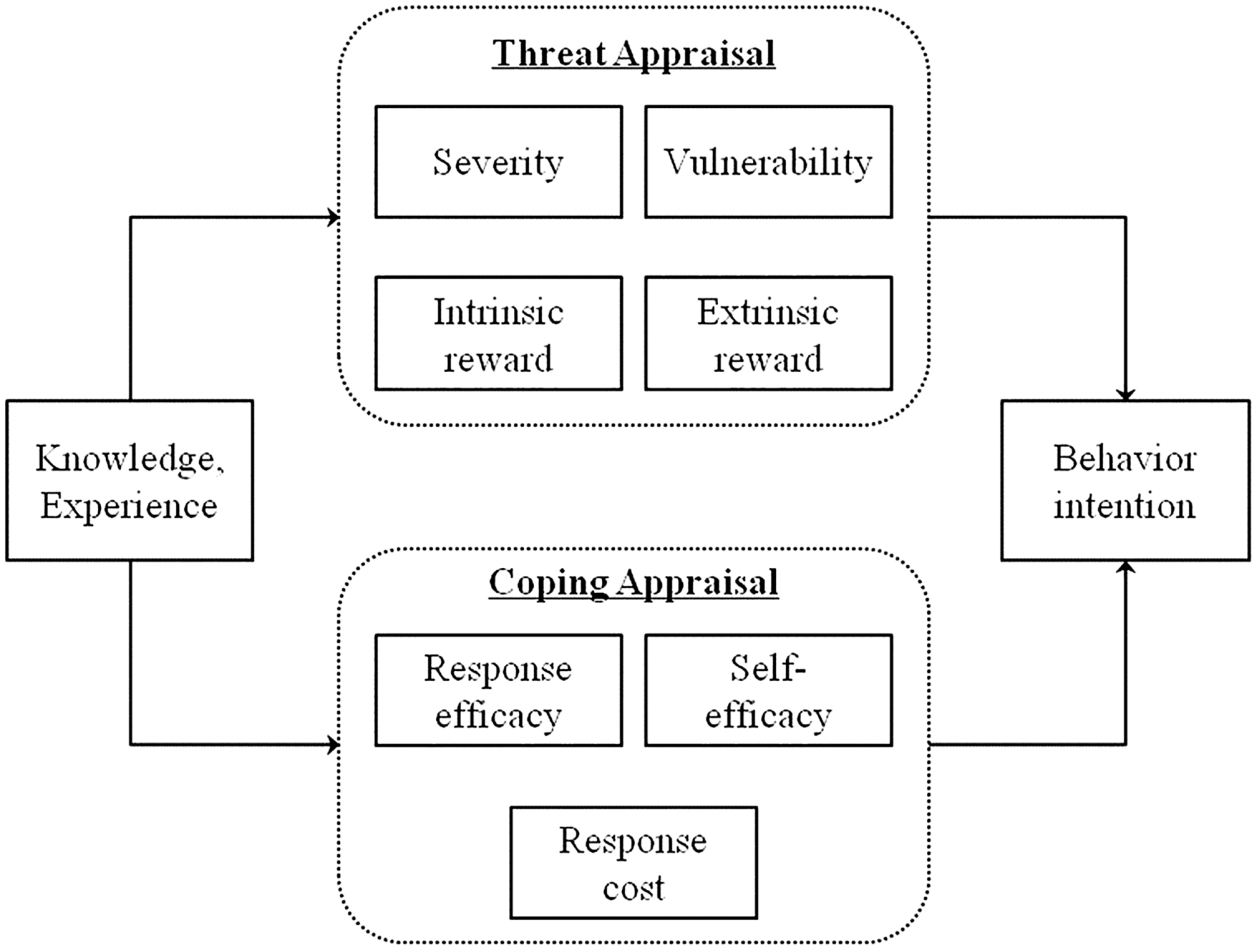
Schematic presentation of the Protection Motivation Theory (PMT) and its seven subconstructs. PMT consists of seven constructs organized as two pathways (i.e. the threat appraisal pathway and the coping appraisal pathway) linking perceptions to behavior. The threat appraisal pathway consists of four constructs in two groups (severity and vulnerability, intrinsic rewards and extrinsic rewards). Coping appraisal pathway consists of three subconstructs in two groups (response efficacy and self-efficacy, response costs).

A review of intervention research literature indicated that the Protection Motivation Theory (PMT) [33]–[35] has been frequently used in HIV prevention research [36]–[38]. The PMT was first introduced by Rogers [33] to understand mechanisms by which people adopt protective behaviors to reduce perceived threat. This model has been revised several times with improved capacity in predicting health-related behaviors [39]–[43]. As a social cognitive model, PMT consists of seven constructs organized as two pathways linking perceptions to behavior (Figure 1). (1) *The threat appraisal pathway* - an evaluation of a person's perception of the threat of certain behaviors or diseases; and (2) *The coping appraisal pathway*- an evaluation of a person's ability to copy with the threat. The threat appraisal pathway consists of four constructs in two groups, with one group assessing perceived threat using two subconstructs (severity, and vulnerability) and another group assessing the perceived rewards also using two subconstructs (i.e. intrinsic rewards and extrinsic rewards). Coping appraisal pathway consists of three subconstructs in two groups, with one group assessing perceived efficacy with two subconstructs (i.e. response efficacy and self-efficacy) and another assessing perceived costs (i.e. response costs).

The PMT may be an alternative to the KAP, because it integrates cognitive process with information, knowledge, attitudes to behavior intentions and further to behaviors [39].The objective of this study was to investigate the applicability of the PMT in understanding the *schistosomiasis*-related knowledge, experiences, attitudes and behavior among young students in China. We hypothesize that *schistosomiasis* knowledge and awareness of *schistosomiasis*, prior exposure to snail-conditioned water, and engagement in protective behaviors will be associated with the PMT constructs, while PMT constructs will predict intentions to engage in protective behaviors. Our purpose is to provide new data supporting theory-based and more effective behavioral interventions for primary prevention of the *schistosomiasis* epidemic.

## Materials and Methods

### Ethics statement

The survey protocol was approved by Institutional Review Board at Wuhan University, China. The written consent was obtained from all students who participated in the study and their parents or guardians.

### Participants and sampling

Data were collected between September and October 2013. Participants were students attending middle school at grade 1 through grade 3. Schools were selected from a high epidemic rural area in Hubei Province, China. Hubei is located in central China, it represents one of the provinces with the highest incidence rates (1.01%) of *schistosomiasis* and the largest number of at-risk population totaling 9.78 million [44]. Data from Hubei Provincial Center for Disease Control and Prevention (CDC) indicate that the number of middle school students with *schistosomiasis* accounts for 51.87% of the total infections [44].

Participants were sampled using a three-step stratified cluster sampling method. In step I: The total 41 epidemic counties in Hubei Province were divided into two strata, stratum one consisting of 19 counties with stool positive rates 1–5%, and stratum two consisting of 22 counties with stool positive rates <1% [45]. Two counties were randomly selected from each stratum, yielding a total of four counties. In step II: two townships were randomly selected from each of the four selected counties in the Step I, yielding a total of eight townships. Within each of the eight selected township, one school with middle school students were randomly selected and invited to participate. In step III: Students in middle school grades 1 to 3 in the participating schools were randomly sampled by class with three classes per grade, yielding a total of 72 classes covering grades 1 to 3 from all the eight participation schools.

All the students in the sampled classes were invited to participate and no student refused to participate. Among the total 2,796 participants who provided data, 274 (9.80%) who responded negatively to the question, “Do you know what is *schistosomiasis*?” were excluded. Among the remaining 2,522 participants, 284 (10.20%) were excluded because of missing data on key variables (age, gender, perceptions of harm from *schistosomiasis*), yielding a final sample of 2238 (80.0%). There was no significant difference in the key study variables among those who were excluded and those included in the study.

### Data collection

Access to each sampled school was obtained from the school administration with the assistance of the local Anti-Schistosomiasis Stations in the sampled county. Data were collected in the classroom settings using the pencil-paper questionnaires. We developed the questionnaire specifically for this study. The developed questionnaire was pilot-tested before it was finalized for the data collection. It took approximately 20 minutes for most students to complete the survey.

The survey was administrated by eight trained data collectors. These data collectors were graduate students from School of Public Health, Wuhan University. To reduce data-collector bias, these graduate students received two-day training. To facilitate completion of the questionnaire, the eight trained data collectors each were assisted by one staff from the local county Anti-Schistosomiasis Stations who was familiar and had good working relationship with the sampled school and students. The staffs from the local Anti-Schistosomiasis Station were responsible to gather the students in a designate classroom. The trained data collectors collected the parental permits and students' informed consents first and then distributed the questionnaires to individual students. The survey was anonymous and confidential. The students were asked to complete the questionnaires independently. They were also allowed to skip questions they did not want to answer. After a student completed the survey, he or she was instructed to hand in the questionnaire to the data collector.

### Measures

#### Knowledge of *schistosomiasis*

Knowledge of *schistosomiasis* was assessed using an 8-item scale. These items assess whether a student knows if humans can be infected with *schistosomiasis* by making contact with snail-conditioned water, whether they knew the routes of transmission, intermediate host, animals that may be infected, the negative health consequence of chronic and advanced *schistosomiasis*, and children's infection. Cronbach's alpha of the knowledge scale was 0.66. Participants earned one point score for each correct answer to the 8 questions and the total score was computed such that larger scores indicated higher levels of knowledge regarding *schistosomiasis* infection.

#### Awareness of *schistosomiasis* and exposure to snail-conditioned water

Participants were asked how frequently they thought about *schistosomiasis* infection before they were exposed to snail-conditioned water in the past 3 months (awareness), responses were coded as (0 = never, 1 = occasionally, 2 = often, and 3 = always). Frequency of exposure to snail-conditioned water in the past 3 months was assessed as: 0 = never if reported “never contacted with snail-conditioned water in the past 3 months”; 1 = less than once a month if reported exposure of “less than one time per month”; 2 = monthly if exposed “1–3 times per month”, and 3 = weekly if exposed “once a week or more”.

#### Previous protective behaviors and future behavioral intentions

Participants were asked how frequently they took preventive measures for *schistosomiasis* (0 = never, 1 = occasionally, 2 = often, and 3 = every time) when they made contact with an infested water in the past 3 months and the past 6 months (protective behavior). Participants were also asked to rate their intentions to engage in protective behaviors against *schistosomiasis* in the next 3 months and next 12 months (behavior intention) using two questions. (1) “I am sure I will not make contact with snail-conditioned water in the next three months and next 12 months.” and (2) “If I have to make contact with snail-conditioned water in the next 3 months and next 12 months, I am sure I will take protective measures.” A 5-point Likert scale was used to assess the two questions with options of 1 = very unlikely to 5 = very likely. Mean scores of the two questions were computed for modeling analysis.

#### The *Schistosomiasis* PMT Scale

We developed the *Schistosomiasis* PMT Scale for this study through team effort. With the coordination of the Principal Investigator Shiyue Li, team members proposed a number of items for each of the seven PMT subconstructs through literature review and brainstorming. Group discussion was followed to select items. When developing the PMT scale, we considered (a) face validity – different items for a subcontract must all be directly related to that construct; (b) balanced items by subconstract –similar number of items for each of all the subconstructs; (c) maximization of Chronbach alpha coefficient as a reliability measure.

The final version of PMT scale consists of 20 items assessing the seven PMT subconstructs. (1) The *vulnerability* was defined as perceived likelihood to be infected by schistosomiasis and was assessed using three items (Cronbach α = 0.82). (2) The *severity* was defined as perceived negative consequences from *schistosomiasis* and was measured with three items (Cronbach α = 0.48). (3) The *intrinsic rewards* was defined as perceived physical and psychological benefits from playing with (snail-conditioned) water and was assessed using three items (Cronbach α = 0.75). (4) The *extrinsic rewards* was defined as perceived social benefits from playing with (snail-conditioned) water, and was assessed using three items (Cronbach α = 0.79). (5) The *response efficacy* was defined as the perceived effectiveness of preventive measures in protecting a person from *schistosomiasis* infection, and was assessed using three items (Cronbach α = 0.63). (6) The *self- efficacy* was defined as personal beliefs in one's own ability to adopt specific protective behaviors against *schistosomiasis* and was assessed using three items (Cronbach α = 0.71). And, (7) The *response costs* was defined as perceived costs incurred by adapting any protective behaviors, and was assessed using two items (Cronbach α = 0.69).

The 20 individual PMT items were assesses using the 5-level response scale with 1 = “definitely agree” to 5 = “definitely disagree”. We purposefully put the negative responses before the positive responses to avoid cohesive answers. Item scores were re-coded such that higher scores indicating more agreement to the individual statements. Mean scores were computed for individual subconstructs for statistical analyses.

#### Demographic variables

Demographic data included chronological age (in years), gender (male and female), school grade (grade 1, grade 2, grade 3), if they were a single child (yes/no), parental education (middle school or less, high school, college or more). In addition to describing the study sample, these variables were used in multivariate analysis.

### Data processing and statistical analysis

Survey data were manually entered into a computer after quality check using the EpiData software. The double-entry protocol was imposed to minimize data-entry errors. Discrepancies from double entries were resolved by consulting the hardcopies of the originally completed questionnaires. Descriptive statistics were used to summarize the sample characteristics. Cronbach alpha was computed to assess the reliability of the PMT subconstructs and other scale measures. Pearson correlation coefficients were computed to assess the relationship among the variables for structural equation modeling analysis. A path modeling approach was used to assess the proposed structural relationship among the PMT subconstructs and other variables. In conducting the path modeling analysis, data-model fitting was assessed using the following four indices: GFI (>0.9), CFI (>0.9), RMSEA (<0.05) and Chi-square/df (<2) [41]. Statistical analyses were completed using the software SAS version 9.2 (SAS Institute Inc. Cary, NC).

## Results

### Sample characteristics

The demographic characteristics of the study sample are displayed in Table 1. Among the total 2,238 students, 1,138 (51%) were male, 31% were in grade one, 37% were grade two, 32% were in grade three, 54% reported that they were a single child in their family. Mean age of the sample was 13 years (SD = 1.1). Most participants reported their parental and maternal educational level to be middle school level, 66% and 62% respectively.

**Table 1:** Characteristics of the study sample.

Variables	Male	Female	Total
**Total, n (%)**	1138 (50.85)	1100 (49.15)	2238 (100.00)
**Chronological age**			
Mean (SD)	13.22 (1.12)	13.03 (1.07)	13.13 (1.10)
**School grade, n (%)**			
Year 1	359 (31.55)	339 (30.82)	698 (31.19)
Year 2	415 (36.46)	410 (37.27)	825 (36.86)
Year 3	364 (31.99)	351 (31.91)	715 (31.95)
**If single child, n (%)**			
Yes	702 (61.69)	500 (45.45)	1202 (53.71)
No	436 (38.31)	600 (54.55)	1036 (46.29)
**Father's education**			
Primary or less	160 (14.06)	160 (14.55)	320 (14.30)
Middle school	742 (65.20)	719 (65.36)	1461 (65.28)
High school or more	236 (20.74)	221 (20.09)	457 (20.42)
**Mother's education**			
Primary or less	223 (19.60)	241 (21.91)	464 (20.73)
Middle school	705 (61.95)	672 (61.09)	1377 (61.53)
High school or more	210 (18.45)	187 (17.00)	397 (17.74)

### Knowledge and awareness of *schistosomiasis* and exposure to snail-conditioned water

The mean score of *schistosomiasis* knowledge is 6.63 (*SD* = 1.59) with no significant gender differences (*t* = 0.97, *p* = 0.33; Table 2). Approximately 70% of participants reported they were always aware of *schistosomiasis* before exposure to the epidemic water. Only 6% of the participants reported frequency of weekly or monthly prior exposure to snail conditioned water. Finally, 74% of participants reported that they engaged in *schistosomiasis* protective behaviors every time when making contact with snail-conditioned water in the past three months.

**Table 2:** Knowledge and awareness of *schistosomiasis* and exposure to snail-conditioned water.

Items	Male	Female	Total
***Schistosomiasis*** ** knowledge**			
Scale score, Mean (SD)	6.66 (1.62)	6.59 (1.57)	6.63 (1.59)
**Awareness of ** ***schistosomiasis*** ** before exposure to epidemic water, n (%)**			
Never	158 (13.88)	98 (8.91)	256 (11.44)
Occasionally	170 (14.94)	120 (10.91)	290 (12.96)
Often	81 (7.12)	55 (5.00)	136 (6.07)
Always	729 (64.06)	827 (75.18)	1556 (69.53)
**Frequency of exposure to snail-conditioned water, last 3 months, n (%)**			
Weekly	46 (4.04)	19 (1.73)	65 (2.90)
Monthly	38 (3.34)	29 (2.63)	67 (3.00)
Less than once a month	97 (8.53)	45 (4.09)	142 (6.34)
Never	957 (84.09)	1007 (91.55)	1964 (87.76)
**Frequency of exposure to snail-conditioned water, last 6 months, n (%)**			
Weekly	45 (3.95)	46 (4.18)	91 (4.07)
Monthly	76 (6.68)	58 (5.27)	134 (5.98)
Less than once a month	187 (16.44)	152 (13.82)	339 (15.15)
Never	830 (72.93)	844 (76.73)	1674 (74.80)
**Engaging in protective behavior, last 3 months, n (%)**			
Never	165 (14.50)	113 (10.27)	278 (12.42)
Occasional	104 (9.14)	83 (7.55)	187 (8.36)
Often	71 (6.24)	42 (3.82)	113 (5.05)
Every time	798 (70.12)	862 (78.36)	1660 (74.17)
**Engaging in protective behaviors, last 6 months, n (%)**			
Never	159 (13,97)	119 (10.82)	278 (12.42)
Occasional	129 (11.34)	84 (7.64)	213 (9.52)
Often	86 (7.55)	46 (4.18)	132 (5.90)
Every time	764 (67.14)	851 (77.36)	1615 (72.16)

### Intention to take protective measures

As shown in Table 3, approximately 6.71% and 7.10% of respondents reported that they are either unlikely or very unlikely to avoid contacting with snail conditioned water in the next 3 and 12 months, while 6.84% and 7.77% reported that they are either unlikely or very unlikely to use protection if they make contact with a snail-conditioned water in the next 3 and 12 months respectively.

**Table 3:** Intention to engage in *schistosomiasis* protective behaviors.

Behavior intentions	Male	Female	Total
Short-term (3 months) intention			
**Likelihood to avoid contacting with snail-conditioned water**			
Very unlikely	45 (3.95)	38 (3.45)	83 (3.71)
Unlikely	37 (3.26)	31 (2.82)	68 (3.04)
Don't know/unsure	240 (21.09)	173 (15.73)	413 (18.45)
Likely	197 (17.31)	197 (17.91)	394 (17.61)
Very likely	619 (54.39)	661 (60.09)	1280 (57.19)
**Likelihood to use protection if having to contact with snail-conditioned water**			
Very unlikely	49 (4.31)	33 (3.00)	82 (3.66)
Unlikely	32 (2.81)	39 (3.55)	71 (3.18)
Don't know/unsure	272 (23.90)	197 (17.91)	469 (20.95)
Likely	247 (21.70)	243 (22.09)	490 (21.90)
Very likely	538 (47.28)	588 (53.45)	1126 (50.31)
Long-term (12 months) intention			
**Likelihood to avoid contacting with snail-conditioned water**			
Very unlikely	48 (4.22)	36 (3.27)	84 (3.75)
Unlikely	41 (3.60)	34 (3.09)	75 (3.35)
Don't know/unsure	301 (26.45)	226 (20.55)	527 (23.55)
Likely	210 (18.45)	184 (16.73)	394 (17.61)
Very likely	538 (47.28)	620 (56.36)	1158 (51.74)
**Likelihood to use protection if having to contact with snail-conditioned water**			
Very unlikely	53 (4.66)	42 (3.82)	95 (4.24)
Unlikely	45 (3.95)	34 (3.09)	79 (3.53)
Don't know/unsure	292 (25.66)	223 (20.27)	515 (23.01)
Likely	221 (19.42)	227 (20.64)	448 (20.02)
Very likely	527 (46.31)	574 (52.18)	1101 (49.20)

### Psychometric characteristics of the *Schistosomiasis* PMT scale

The mean and standard deviation of each item in *Schistosomiasis* PMT scale are presented in Table 4. The item to total correlations ranged from 0.06 to 0.50, Cronbach's alpha coefficient >0.7 among four of the seven PMT subconstructs and Cronbach's alpha coefficient = 0.76 for the overall PMT Scale.

**Table 4:** *Schistosomiasis* PMT scale.

*Schistosomiasis* PMT scale	Mean (SD)	Item-total r	Alpha
***Severity***			0.48
1. *Schistosomiasis* causes big damage to people's health.	4.73 (0.60)	0.26	
2. My whole family will suffer if I am infected with *schistosomiasis*.	2.87 (1.22)	0.12	
3. I will become hopeless for the rest of my life if I am infected with *schistosomiasis*.	2.43 (1.16)	0.07	
***Vulnerability***			0.82
4. I will get infected if I contact snail-conditioned water while assisting my parents to work.	4.32 (0.84)	0.38	
5. I will get infected if I play with or swim in snail-conditioned water.	4.39 (0.81)	0.42	
6. If I wade through water or walk in wetland in snail-conditioned areas without protection, I will get infected.	4.36 (0.83)	0.38	
***Intrinsic Reward***			0.75
7. It is very enjoyable for me to play in water outside.	2.05 (1.26)	0.47	
8. It is convenient to wash my hands and feet in rivers and ponds.	1.99 (1.25)	0.50	
9. It will be more convenient to assist parents' work or play in snail-conditioned water without protective measures.	2.02 (1.31)	0.44	
***Extrinsic Reward***			0.79
10. Playing in outdoor water with classmates is good for making friends and for strengthening friendship.	1.90 (1.10)	0.47	
11. Washing my hands and feet or clothes in river and ponds will save tap water, save money for my family.	1.93 (1.14)	0.44	
12. It will save money if assisting parents' work or playing in water without protection (water shoes, protective gels).	1.72 (1.01)	0.46	
***Response Efficacy***			0.63
13. I will never be infected if I do not play in snail-conditioned water.	2.76 (1.14)	0.06	
14. I will never get infected if I do not assist parents' work in snail-conditioned water.	2.80 (1.12)	0.06	
15. I will never get infected if I use good protection measures before contacting with snail-conditioned water.	4.16 (0.96)	0.19	
***Self-Efficacy***			0.71
16. I can control myself not to go and play in snail-conditioned water.	4.20 (1.02)	0.26	
17. I can definitely say “no” even if my friends invite me to play in snail-conditioned water.	4.47 (0.92)	0.40	
18. I can definitely say “no” even if my parents or teachers ask me to do things that may involve snail-conditioned water.	4.38 (0.98)	0.41	
***Response Cost***			0.69
19. My friends may tease me if I refuse to go out with them to play in snail-conditioned water.	2.15 (1.28)	0.36	
20. My parents will scald me if I worry about *schistosomiasis* and refuse to do things in snail-conditioned water.	1.86 (1.15)	0.33	

### Association between the PMT constructs and behavior measures

Data in Table 5 indicated that (1) all the four predictor variables (knowledge, awareness, prior exposure and engagement in protective behaviors) were intercorrelated at p <0.05 or p<0.01 level. (2) All the seven PMT subcontucts were significantly intercorrrelated with each at p<0.05 or 0.01 level with one exception between severity and extrinsic rewards (p>0.05). (3) Five of the seven PMT subconstructs (e.g., vulnerability, intrinsic rewards, extrinsic rewards, self-efficacy and response efficacy) were significantly associated with the four predictor variables (e.g., knowledge, awareness, prior exposure to snail-contained water, and prior engagement in protective behavior). Among the remaining two subcontracts, severity was significantly associated with prior engagement in protective behavior and future behavior intentions, response efficacy was significantly associated with knowledge and future behavior intentions. (4) The seven PMT subconstructs and the four predictor variables were all significantly associated with intention to engage in protective behaviors (p<0.01 for all).

**Table 5:** Correlations of the *schistosomiasis* PMT subconstructs with knowledge and behavioral measures.

Variable	Mean (SD)	2	3	4	5	6	7	8	9	10	11	12
1.Severity	3.34 (0.72)	0.16**	−0.06**	0.01	0.09**	0.06**	0.01	0.03	0.002	0.04	0.04*	0.08**
2.Vulnerability	4.35 (0.71)		−0.23**	−0.19**	0.09**	0.25**	−0.19**	0.28**	0.14**	0.07**	0.15**	0.20**
3.Intrinsic rewards	2.02 (1.04)			0.55**	0.05*	−0.25**	0.29**	−0.18**	−0.15**	−0.14**	−0.21**	−0.12**
4.Extrinsic rewards	1.85 (0.91)				0.11**	−0.22**	0.37**	−0.19**	−0.13**	−0.14**	−0.17**	−0.12**
5.Response efficacy	3.24 (0.82)					0.11**	0.04*	0.08**	0.03	−0.001	0.04	0.13**
6.Self efficacy	4.35 (0.78)						−0.19**	0.11**	0.16**	0.15**	0.20**	0.40**
7.Response cost	2.01 (1.06)							−0.15**	−0.11**	−0.13**	−0.15**	−0.12**
8.Knowledge	6.63(1.59)								0.09**	0.05*	0.08**	0.10**
9.Awareness consideration	2.34 (1.08)									0.25**	0.46**	0.15**
10.Prior exposure	2.79 (0.63)										0.31**	0.12**
11.Protective behavior	2.41 (1.07)											0.17**
12.Behavior intention	4.17 (0.97)											

**Note**:

**p<0.01,

*p<0.05.

### Results from the structural equation modeling analysis

**Figure 2:**
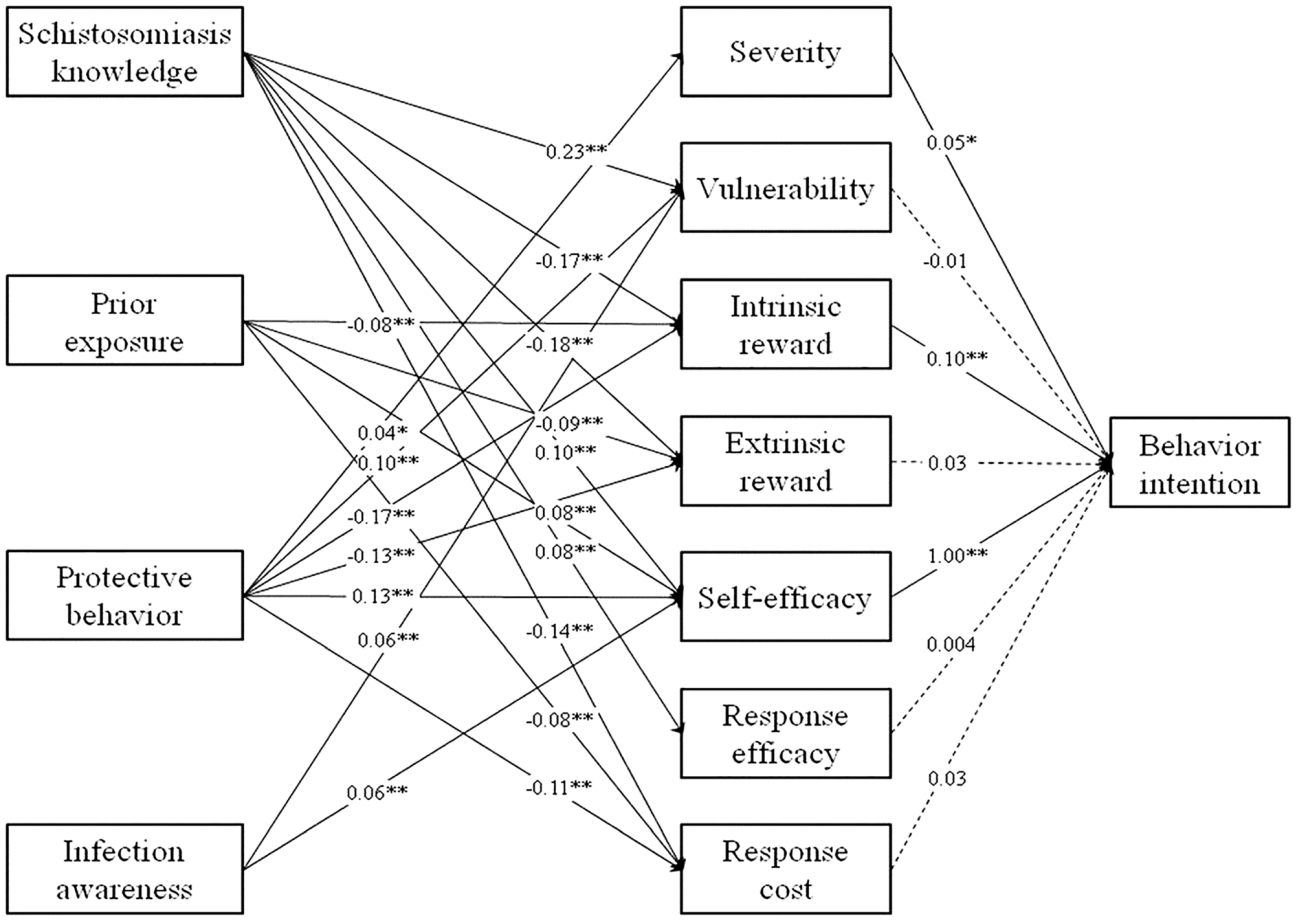
Structural equation modeling of *schistosomiasis* knowledge, prior exposure, awareness, protective behavior, *schistosomiasis* PMT constructs and behavior intention. The data-model fit indices: GFI = 0.98, CFI = 0.93, RMSEA = 0.06, Chi-square/df = 8.6. **: p<0.01 and *: p<0.05.

Results in Figure 2 partially support the proposed structural association that *schistosomiasis* knowledge, awareness of *schistosomiasis* infection, prior exposure to snail-contained water and engagement in protective behavior each are associated with PMT constructs, which in turn are associated with behavior intentions. Our data fit the hypothesized model well (GFI = 0.98, CFI = 0.93, RMSEA = 0.06, Chi-square/df = 8.6). The estimated parameters indicate that the four predictor variables each were significantly associated with at least two of the seven PMT subconstracts at p<0.01 level. Among the four predictor variables, two (knowledge and engagement in protective behaviors) were associated with six of the seven PMT subconstructs. Among the seven PMT subconstructs, three (severity, intrinsic rewards and self-efficacy) were significantly associated with intentions to engage in protective behavior.

In addition to assess behavior intentions in the next 3 months, longer-term intention was also assessed using the same SEM approach. The results were quite similar to those reported in Figure 2.

## Discussion

As a social cognitive conceptual framework, the Protection Motivation Theory (PMT) has been widely used in predicting health behaviors [46], [47] and in guiding research to devise intervention programs for purposeful behavior change [36]–[38]. This is the first study to investigate the applicability of PMT in understanding the *schistosomiasis* protective behavior among middle school students in rural China. This *Schistosomiasis* PMT Scale has adequate reliability with Cronbach's alpha coefficients greater the 0.7 (considered as good) for the overall scale and for most of its subconstracts. The PMT Scale also has adequate validity because in addition to predicting behavioral intentions, the seven PMT subconstructs were associated with all the predictor variables used in this analysis, including previous use of protective measures, knowledge of *schistosomiasis*, awareness of *schistosomiasis* infection, and previous exposure to risk factors.

Despite rapid economic development, *schistosomaisis* remains a public health challenge in many epidemic regions, particularly the rural areas in China and many other developing countries [48]–[50]. Findings of this study provide evidence supporting the significance of the PMT, a US-developed theory in guiding behavioral research and intervention, for *schistomaisis* control in China. To our knowledge, PMT theory can be considered as an expansion of Knowledge-Attitude-Practice (KAP) Theory to encompass such components as cognitive appraisal of the risks of *schistomaisis* infection, and benefits from using protection and self-coping strategies. In addition to information-based education and skills, PMT emphasizes training for decision making by assessing the risks of diseases and benefits from protection, and motivate participants to take protective actions against diseases.

Consistent with the PMT theory, results from correlation and structural equation modeling analysis of this study indicate that *schistosomiasis* knowledge, awareness of infection, prior exposure to snail-contained water, and engagement in protective behaviors are associated with various PMT constructs. This finding suggests that in addition to the cognitive components related to PMT, intervention programs may benefit by considering previous knowledge, experience and cognition of *schistosomaisis* [51]. Relative to students with previous knowledge and protective experience, more efforts may be needed to encourage naive youth to use protection against *schistosomaisis*.

Another key finding of this study is that both the PMT threat appraisal pathway (e.g., the severity and intrinsic rewards) and the PMT coping appraisal pathway (e.g., self-efficacy) are associated with intention to engage in protective behaviors. The role of coping appraisal (particularly self-efficacy) has been reported in intervention studies focused on HIV risk behavior prevention [36]–[38], and there is a lack of data supporting the role of the threat appraisal. One reason for our findings is that compared to HIV related behaviors (e.g., sexual initiation, use of a condom during sex, use of alcohol and drugs), *schistosomaisis*-related behavior are less likely to be affected by situational and emotional factors. Students may have adequate time to think about the threats of *schistosomasis* and the pros and cons of using or not using protection. If this is the case, we can anticipate that a PMT-based intervention could be more effective to encourage protective behaviors against *schistosomaisis* than HIV if both the threat appraisal and the coping appraisal are emphasized.

From an applied perspective, the findings from the study suggest that several variables (i.e. severity, intrinsic rewards and self-efficacy), especially self-efficacy, should be targeted in *schistosomaisis* intervention programs for adolescents in order to increase their behavior intentions. First, adolescents' believe in their ability to avoid contact with snail-conditioned water despite barriers (i.e. self-efficacy) should be improved through providing positive coping messages to enhance their confidence regarding protective behaviors and their belief that protective behaviors provides health benefits. Second, realistic information about disease severity should be concentrated on to increase their perceived level of fear about the disease for promotion acceptance of the proposed adaptive behavior or intention.

This study has some limitations. First, data used for this study was collected through a cross-sectional survey. Therefore the observed relationship between PMT subconstructs and other variables need to be verified through longitudinal studies. Second, although overall the *Schistosomaisis* PMT scale has adequate reliability, the Chronbach's alpha coefficients were less than satisfactory for two PMT subconstructs severity (alpha = 0.48) and response efficacy (alpha = 0.63). These relatively low alpha coefficients suggest the need for further work to improve the reliability of these two measures. Despite the limitations, this study is the first to systematically test the utility of a US-developed behavioral theory in describing *schistosomaisis* behavior among middle school students in China. Findings from this study add novel data supporting more effective *schistosomiasis* prevention interventions.

## Supporting Information

Checklist S1 STROBE Checklist.
(DOC)
